# Dopamine Receptor Activation Is Required for GABAergic Spike Timing-Dependent Plasticity in Response to Complex Spike Pairing in the Ventral Tegmental Area

**DOI:** 10.3389/fnsyn.2018.00032

**Published:** 2018-09-21

**Authors:** Ludovic D. Langlois, Matthieu Dacher, Fereshteh S. Nugent

**Affiliations:** Department of Pharmacology, F. Edward Hebert School of Medicine, Uniformed Services University of the Health Sciences, Bethesda, MD, United States

**Keywords:** ventral tegmental area, VTA, spike-timing dependent plasticity, STDP, synaptic plasticity, long-term depression, LTD, GABAergic synapses

## Abstract

One of the most influential synaptic learning rules explored in the past decades is activity dependent spike-timing-dependent plasticity (STDP). In STDP, synapses are either potentiated or depressed based on the order of pre- and postsynaptic neuronal activation within narrow, milliseconds-long, time intervals. STDP is subject to neuromodulation by dopamine (DA), a potent neurotransmitter that significantly impacts synaptic plasticity and reward-related behavioral learning. Previously, we demonstrated that GABAergic synapses onto ventral tegmental area (VTA) DA neurons are able to express STDP (Kodangattil et al., 2013), however it is still unclear whether DA modulates inhibitory STDP in the VTA. Here, we used whole-cell recordings in rat midbrain slices to investigate whether DA D1-like and/or D2-like receptor (D1R/D2R) activation is required for induction of STDP in response to a complex pattern of spiking. We found that VTA but not Substantia nigra pars compact (SNc) DA neurons exhibit long-term depression (LTD_GABA_) in response to a combination of positive (pre-post) and negative (post-pre) timing of spiking (a complex STDP protocol). Blockade of either D1Rs or D2Rs prevented the induction of LTD_GABA_ while activation of D1Rs did not affect the plasticity in response to this complex STDP protocol in VTA DA neurons.Our data suggest that this DA-dependent GABAergic STDP is selectively expressed at GABAergic synapses onto VTA DA neurons which could be targeted by drugs of abuse to mediate drug-induced modulation of DA signaling within the VTA, as well as in VTA-projection areas, thereby affecting reward-related learning and drug-associated memories.

## Introduction

In the past few decades, synaptic plasticity has emerged as an important candidate mechanism for drug-induced changes in reward-related neural circuits where addictive drugs usurp synaptic mechanisms underpinning reward/motivational or aversive behavioral learning to alter dopamine (DA) signaling from the ventral tegmental area (VTA), a critical brain region involved in reward and motivation (Pignatelli and Bonci, 2015; Langlois and Nugent, 2017). Drug-induced synaptic plasticity has attracted considerable interest in studies of drug addiction because strong and durable memories associated with drug experience are demonstrated to promote compulsive drug taking, craving and relapse.

Experimental synaptic plasticity can be induced using traditional induction paradigms as well as spike-timing-dependent plasticity (STDP) protocols. STDP is considered a physiological form of plasticity that relies on the relative timing of neuronal activity (Feldman, 2012). Specifically, STDP at glutamatergic and GABAergic synapses within DA-related neural circuits likely presents a critical synaptic learning rule for encoding natural reward-related learning and memory (Pawlak et al., 2010; Langlois and Nugent, 2017; Foncelle et al., 2018). The Hebbian learning rules of STDP dictates that long-term potentiation (LTP) is induced when presynaptic activity precedes postsynaptic spiking (pre-post spiking, positive timing), whereas reversing the order induces long-term depression (LTD, post-pre spiking, negative timing; Dan and Poo, 2006; Caporale and Dan, 2008). Recently, it has become clear that GABAergic synaptic plasticity in addition to the widely studied glutamatergic plasticity is targeted by drugs of abuse and drug-induced modulation of this plasticity could critically influence DA neuronal activity and DA release in VTA projection areas, as well as in local VTA microcircuits (Langlois and Nugent, 2017). We previously demonstrated that GABAergic synapses onto VTA DA neurons are able to exhibit a Hebbian heterosynaptic STDP where near-coincident and correlated activities of presynaptic glutamatergic neurons with postsynaptic DA neurons result in expression of LTP and LTD (that we call LTP_GABA_ and LTD_GABA_). More importantly, we found that GABAergic synapses are predisposed to undergo LTD_GABA_ in response to spike trains (a complex STDP protocol that includes bursts of a combination of pre-post and post-pre spiking; Kodangattil et al., 2013). Somatodendritically released DA within the VTA acts on DA D2-like receptors (D2Rs) and presents an important mechanism for controlling the excitability of DA neurons (Beckstead et al., 2004). There is also an emerging neuromodulatory role for DA in STDP (Pawlak et al., 2010). In fact, our previous study (Dacher and Nugent, 2011) demonstrated that the induction of LTD in response to a traditional LTD pairing protocol (a combination of low frequency stimulation (LFS) with modest depolarization) is dependent on D2R activation. Here we sought to explore the potential effects of pharmacological manipulation of DA transmission within the VTA on the induction of GABAergic STDP in response to complex STDP protocols. We found that DA action through either D1Rs or D2Rs is necessary and sufficient for the induction of LTD_GABA_ in VTA DA neurons and that this DA-dependent plasticity is limited to GABAergic synapses in the VTA. Given that drugs of abuse increase DA neurotransmission within the VTA (Bradberry and Roth, 1989; Klitenick et al., 1992; Campbell et al., 1996; Rahman et al., 2003), our present findings provide an inhibitory synaptic mechanism by which drug-induced alteration of local VTA DA signaling could affect DA cell excitability and subsequently DA release in VTA DA circuits.

## Materials and Methods

Brain slice preparation and electrophysiological recordings were conducted as described previously from 14 days to 21 days old Sprague-Dawley rats (Dacher et al., 2013; Kodangattil et al., 2013). Briefly, animals were anesthetized using isoflurane and quickly decapitated. The brain was rapidly dissected and placed into ice-cold artificial cerebrospinal fluid (ACSF) containing (in mM): 126 NaCl, 21.4 NaHCO_3_, 2.5 KCl, 1.2 NaH_2_PO_4_, 2.4 CaCl_2_, 1.00 MgSO_4_, 11.1 glucose, 0.4 ascorbic acid, saturated with 95% O_2_/5% CO_2_. Horizontal midbrain slices containing the substantia nigra pars compact (SNc) and VTA were cut (250 μm) and incubated in ACSF during at least 1 h at 34°C. Slices were then transferred into a recording chamber in ascorbic acid-free ACSF at 28°C. All experiments were carried out in accordance with the National Institutes of Health (NIH) guidelines for the care and use of laboratory animals and were approved by the Uniformed Services University Institutional Animal Care and Use Committee. All efforts were made to minimize animal suffering, and to reduce the number of animals used.

GABA_A_ inhibitory post-synaptic currents (IPSCs) were recorded using a patch amplifier (Multiclamp 700B) under infrared-differential interference contrast microscopy. Data acquisition and analysis were performed using DigiData 1440A and pClamp 10 (Molecular Devices, Union City, CA, USA). In all experiments, 6,7-dinitroquinoxaline-2,3-dione (DNQX, 10 μM) and strychnine (1 μM) obtained from Sigma were added to block AMPA- and glycine-mediated synaptic currents, respectively to pharmacologically isolate GABA_A_ IPSCs that were completely blocked by the GABA_A_ receptor antagonist, bicuculline. Paired GABA_A_ IPSCs were evoked using a bipolar stainless steel stimulating electrode placed 200–500 mm rostral to the recording site in the VTA at 0.1 Hz (duration 100 μs, 50 ms inter-stimulation interval) and recorded using KCl containing electrodes and whole-cell voltage-clamp in neurons held at −70 mV. Pipettes were filled with (in mM): 125 KCl, 2.8 NaCl, 2 MgCl_2_, 2 ATP-Na^+^, 0.3 GTP-Na^+^, 0.6 EGTA and 10 HEPES (pH adjusted to 7.28 with KOH, osmolarity adjusted to 275–280 mOsm with sucrose). Stimulation intensity was adjusted to evoke baseline synaptic responses ranged between −200 pA and −800 pA (approximately 50% of maximal responses). The cell input resistance and series resistance were monitored through the experiment and if these values changed by more than 10%, data were not included. The appearance of an Ih current (≥50 pA) in response to stepping cells from −50 mV to −100 mV was used to identify putative SNc/VTA DA neurons. As a standard protocol in our lab including the present study we consistently record from a region of the VTA (in the dorsal and caudal VTA) that is shown to contain mostly nucleus accumbens (NAc)- projecting DA neurons with Ih positivity (Margolis et al., 2006a,b; Zhang et al., 2010). In addition we consistently consider other electrophysiological criterions for identification of DA neurons (AP characteristics and frequency) that are also commonly used to identify putative DA neurons (Johnson and North, 1992; Dacher et al., 2013; Kodangattil et al., 2013).

**Figure 1:**
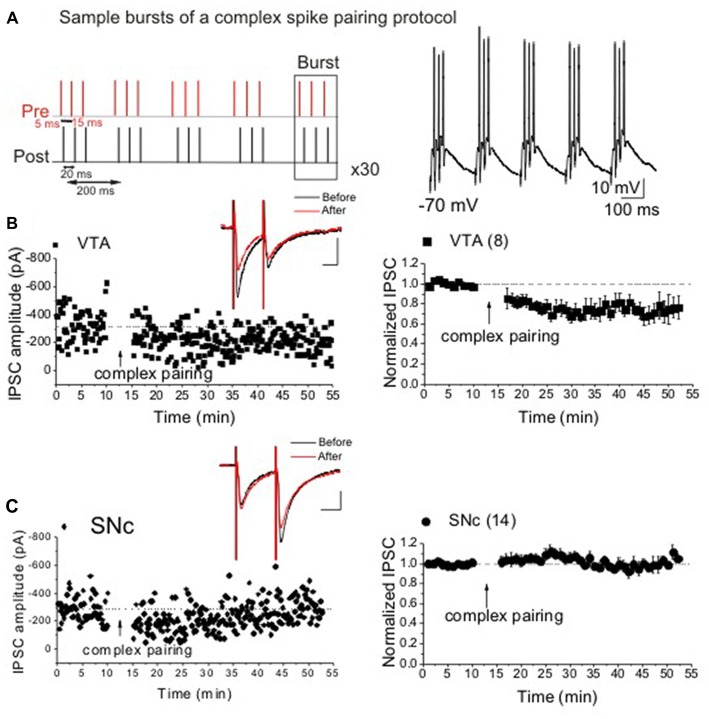
Ventral tegmental area (VTA) but not substantia nigra pars compact (SNc) dopamine (DA) neurons express spike-timing-dependent (STD)-long-term depression (LTD_GABA_) in response to a complex spiking spike-timing-dependent plasticity (STDP) protocol. Panel **(A)** represents sample bursts of the complex spiking protocol for induction of LTD_GABA_. **(B,C)** Single and average experiments showing induction of STDP recorded in Ih^(+)^ (presumably DA) neurons in VTA (filled square symbols) or SNc (filled circle symbols). At the arrow, STDP was induced. Insets: averaged inhibitory postsynaptic currents (IPSCs) before and 25 min after STDP protocol. In this and all figures, 10 consecutive traces from each condition were averaged for illustration as inset. Calibration: 100 pA, 25 ms (VTA: 75 ± 1.1% of pre-STDP values, *F*_(3,22)_ = 7.5, *p* < 0.0001, *n* = 8; SNc: 103 ± 2.4% of pre-STDP values, *F*_(5,70)_ = 1.26, *n* = 14). Values shown throughout figure are the mean ± SEM.

To induce STDP, we first obtained a stable baseline for 10 min and then DA cells were taken to current clamp and received trains of a sub-threshold presynaptic stimulation paired with back propagating action potentials (bAPs/postsynaptic spiking) at 5 Hz. To evoke bAPs, cells were injected with direct somatic currents of 1.5 nA for 5 ms through patch pipettes. STDP protocols consisted of 30 trains of five bursts repeated at 0.1 Hz. To induce LTD_GABA_ using post-pre pairing, each burst was composed of three bAPs at 50 Hz followed by a single presynaptic stimulation (negative timing, −5 ms). A complex STDP protocol was used to induce LTD_GABA_ where each burst composed of three bAPs preceded with three presynaptic stimulations at 50 Hz (the complex pairing protocol included both positive timing, +5 ms and negative timing, −15 ms, Figure 1A). Values are presented as means ± SEM. Statistical significance was assessed using repeated measures ANOVA with significance level of *p* < 0.05. Levels of STDP are reported as averaged IPSC amplitudes for 5 min just before STDP induction compared with averaged IPSC amplitudes during the 5 min period from 25 min to 30 min after protocol. Interleaved control experiments were performed with experiments in which drugs were bath applied. Salts and all drugs were purchased from Sigma-Research Biochemicals International or Tocris Bioscience.

## Results

### VTA but Not SNc DA Neurons Exhibit LTD_GABA_ in Response to a Complex Pairing STDP Protocol

Previously, we showed that the synaptic efficacy of GABAergic synapses onto VTA DA neurons can be bi-directionally modified by pre/post spike pairing in a narrow time window (Kodangattil et al., 2013; Authement et al., 2015). Here, we used a more natural and complex pattern of spiking (Figure 1A) to induce STDP at GABAergic synapses onto midbrain VTA/SNc DA neurons. Consistent with our previous results (Kodangattil et al., 2013), we were able to induce LTD_GABA_ in VTA DA neurons in response to a combination of both positive (+5 ms) and negative (−15 ms) timing (Figure 1B). On the other hand, we found that SNc DA neurons did not exhibit any form of plasticity in response to the same complex STDP protocol (Figure 1C). Given this finding, we only examined the effects of D1R/D2R drugs on the induction of STDP (LTD_GABA_) in VTA DA neurons.

### Induction of LTD_GABA_ in the VTA by STDP Protocols Requires D2R Activation

**Figure 2:**
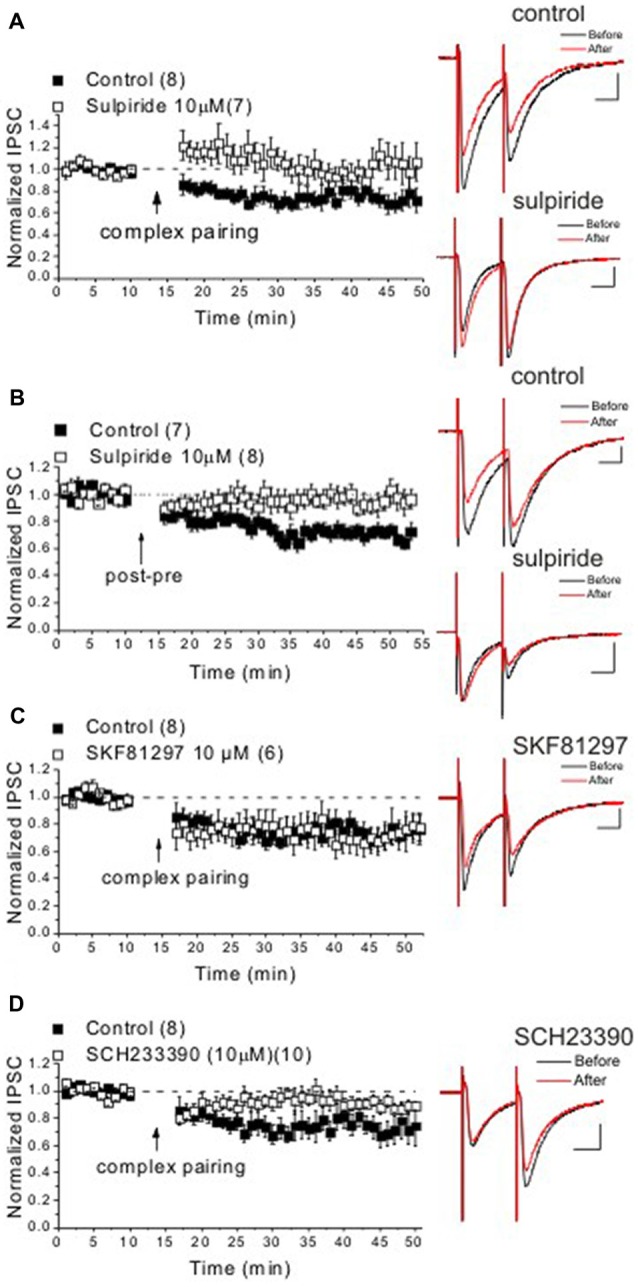
DA D1-like receptor (D1R) and D2R activation is required for induction of LTD_GABA_ in response to the complex STDP protocol. Panels **(A–D)** show average experiments of STDP with sample traces from Ih^(+)^ neurons in response to the complex or post-pre STDP protocols in drug-free artificial cerebrospinal fluid (ACSF; controls, filled square symbols) or drug bath application (open square symbols) experiments. Control LTD_GABA_ group in response to the complex STDP protocol is similar to Figure 1B in **(A,C,D)** Representing the interleaved control experiments conducted with drug-treated slice experiments. **(A)** The D2R antagonist blocked the induction of LTD_GABA_ in response to the complex protocol (sulpiride: 104 ± 1.8% of pre-STDP values, *F*_(4,14)_ = 0.33, *n* = 7). **(B)** Sulpiride prevented the induction of LTD_GABA_ in response to the pre-post protocol while control slices showed robust LTD_GABA_ (control: 69 ± 1.8% of pre-STDP values, *F*_(4,25)_ = 7.01, *p* < 0.0001, *n* = 7; sulpiride: 96 ± 1.5% of pre-STDP values, *F*_(4,32)_ = 0.63, *n* = 8). **(C)** The D1R agonist did not affect the induction of LTD_GABA_ in response to the complex protocol (SKF81297: 75 ± 1.2% of pre-STDP values, *F*_(3,12)_ = 3.7, *p* < 0.05, *n* = 7). **(D)** The D1R antagonist blocked the induction of LTD_GABA_ in response to the complex protocol (SCH23390: 89 ± 1.04% of pre-STDP values, *F*_(4,32)_ = 2.01, *n* = 10). Calibration: 100 pA, 25 ms.

LTD_GABA_ at GABAergic synapses onto VTA DA neurons can also be triggered in response to a traditional LTD pairing paradigm using LFS paired with modest depolarization (Dacher and Nugent, 2011). LTD_GABA_ triggered in response to both LFS-pairing paradigm and STDP protocols is dependent on the postsynaptic scaffolding A-kinase anchoring protein 79/150 (AKAP79/150) signaling complex which selectively controls GABAergic synaptic strength and mediates the opposing effects of protein kinase A (PKA) and calcineurin (CaN) on GABA_A_ receptor trafficking in VTA DA neurons (Dacher and Nugent, 2011; Dacher et al., 2013; Authement et al., 2015). Since LTD_GABA_ in response to the LFS-pairing LTD paradigm is also D2R-dependent and modulated by morphine (Dacher and Nugent, 2011), we tested whether LTD_GABA_ induced by STDP protocols also requires D2R activation. We attempted to induce LTD_GABA_ by a complex STDP protocol or a post-pre STDP protocol as previously described (Kodangattil et al., 2013; Authement et al., 2015) while a D2R antagonist, sulpiride (10 μM), was present in the perfusate throughout the experiment. Sulpiride was able to completely block the induction of LTD_GABA_ in response to the complex STDP protocol (Figure 2A) as well as post-pre STDP protocol (Figure 2B) suggesting that both the pairing and STDP protocols trigger this D2R-dependent LTD_GABA_. It should be mentioned that D2R activation by a D2R agonist results in a rundown in GABAergic IPSCs (a chemical form of LTD) in VTA DA neurons that is dependent on CaN activity upon inhibition of PKA-AKAP150 anchoring (Dacher et al., 2013).

### Induction of LTD_GABA_ by a Complex STDP Protocol Also Requires D1R Activation

The roles of D1Rs and D2Rs have been implicated in induction and modulation of STDP (Pawlak and Kerr, 2008; Shen et al., 2008; Pawlak et al., 2010; Xu and Yao, 2010; Ruan et al., 2014). Although DA via both D1R and D2R may exert opposing effects on STDP where D1Rs enable the induction of LTP and D2Rs mainly favor LTD, the cooperative actions of both DA receptors in induction of LTD of STDP have been shown (Xu and Yao, 2010). Here we tested our hypothesis that D1R activation blocks LTD_GABA_ and reverses the direction of plasticity to promote LTP_GABA_. To test this, we attempted to induce LTD_GABA_ in response to the complex pairing STDP protocol in the presence of a D1R agonist (SKF81297, 10 μM) or a D1R antagonist (SCH233390, 10 μM). While LTD_GABA_ was unaffected in the presence of the D1R agonist (Figure 2C), the D1R antagonist blocked the induction of LTD_GABA_ (Figure 2D) suggesting that D1R activation also facilitates the induction of LTD at GABAergic synapses onto VTA DA neurons.

## Discussion

Here, we have extended our previous studies on GABAergic STDP in VTA DA neurons to investigate how local VTA DA signaling through somatodendritic release of DA during STDP within the VTA would affect STDP. We found that while GABAergic synapses onto VTA DA neurons were able to express LTD_GABA_ in response to a complex STDP protocol, GABAergic synapses onto SNc DA neurons did not show any form of plasticity in response to the same protocol. This suggests that this form of inhibitory plasticity may be selectively expressed at GABA_A_ synapses in the VTA. LTD_GABA_ triggered by our complex STDP protocol or a post-pre STDP protocol in VTA DA neurons was dependent on D2R activation similar to LTD_GABA_ triggered in response to a typical LFS-pairing protocol shown by our group (Dacher and Nugent, 2011). D1R activation seems to commonly facilitate the induction of LTP in response to STDP protocols although it could also contribute to induction of LTD of STDP at the synapse (Pawlak and Kerr, 2008; Zhang et al., 2009; Ruan et al., 2014; Brzosko et al., 2015). Thus, we further examined whether boosting endogenous DA action on D1R activity during STDP induction through bath application of a D1R agonist could reverse the direction of plasticity towards LTP. Not only were we unable to trigger LTP in response to the complex protocol in the presence of D1R agonist, the induction of LTD_GABA_ was completely unaffected ruling out the possibility of masking LTD by a simultaneous induction of a D1R-dependent LTP at these synapses. Interestingly, we observed a blockade of LTD_GABA_ in response to the complex protocol in the presence of D1R antagonist suggesting that local endogenous DA could engage D1Rs to promote this plasticity. The selectivity of induction of GABAergic STDP at GABAergic synapses in the VTA vs. the SNc may not be surprising considering the distinct anatomical and functional populations of VTA and SNc DA neurons (Beier et al., 2015; Shin et al., 2017). Given that VTA DA neurons are found to be more heterogeneous than SNc DA neurons (Margolis et al., 2006b) and the postsynaptic nature of DA-dependent LTD_GABA_ triggered at GABAergic synapses in the VTA (Dacher and Nugent, 2011; Dacher et al., 2013; Kodangattil et al., 2013; Authement et al., 2015), our data suggest that the expression of this STDP as a uniform property of GABA_A_ synapses in the VTA may be due to distinct intrinsic characteristics of the VTA microcircuits that differ from the SNc local circuitry. Consistently, it has been shown that extracellular DA levels released within the VTA and SNc differ as the dendritic release of DA within the VTA is far greater than DA release within the SNc (Rice et al., 1997; Ford et al., 2010). Therefore, we propose that this regional difference in release properties of DA at dendritic locations may underlie the selective expression of STDP in the VTA. Given that activation of either D1Rs or D2Rs by endogenous DA was sufficient to trigger this plasticity and considering different localization of DA receptors in VTA neuronal populations and presynaptic terminals innervating VTA neurons, it remains to be known how and where the activation of either presynaptic or postsynaptic DA receptors mediate this plasticity in the VTA. GABA_A_ synapses onto VTA DA neurons mainly originate from VTA GABAergic neurons (that comprise 30% of VTA neuronal populations) and rostromedial tegmental area (RMTg) neurons (Barrot et al., 2012). D2Rs are mainly expressed postsynaptically on VTA DA neurons to provide an auto feedback inhibition of DA neurons (Beckstead et al., 2007). D2Rs are assumed to be expressed on GABAergic terminals where their activation facilitates the induction of a presynaptic endocannabinoid-mediated LTD at GABAergic synapses onto VTA DA neurons (Pan et al., 2008). We have also shown that inhibition of PKA activity (which is the main downstream signaling mechanism for both D1R and D2R) or disruption of AKAP150-PKA association promotes LTD_GABA_ in response to STDP protocols by favoring CaN activity and endocytosis of GABA_A_ receptors in VTA DA neurons (Authement et al., 2015). Given that D2Rs can inhibit PKA activity and the postsynaptic locus of LTD_GABA_ expression, we assume that D2R activation acts through this signaling pathway to promote LTD_GABA_ in response to complex STDP protocols. Only a small subset of VTA DA neurons express D1Rs (D1/D5; Schilström et al., 2006). DA increases presynaptic release of GABA in the midbrain through D1R activation (Cameron and Williams, 1993). It has been shown that NAc D1R-expressing medium spiny neuronal projections inhibiting VTA DA neurons preferentially make GABA_B_ synapses onto VTA DA neurons while these D1R expressing neurons of NAc inhibit VTA GABAergic interneurons via activating GABA_A_ synapses (Barrot et al., 2012). It will be interesting to test whether the postsynaptic action of DA on D1Rs in VTA DA neurons promotes LTD through modulation of NMDA receptor (Schilström et al., 2006) or GABA_B_ receptor activity originating from D1 expressing NAc (Cameron and Williams, 1993; Kamikubo et al., 2007) in VTA DA neurons. It should be noted that there are several limitations to our study including the young age of the rats, the suboptimal cooler temperature for recordings at 28°C, the use of electrical stimulation rather than optogenetic stimulation of specific afferent inputs to the VTA, and also the complexity of mimicking a natural pattern of neuronal firings in an *in vitro* STDP induction protocol. In fact, a recent study has shown the heterogeneity of distinct GABAergic inputs to VTA DA neurons where only GABAergic inputs arising from the VTA GABAergic interneurons show short-term plasticity (Polter et al., 2018). In sum, we demonstrated that synaptic actions of DA within the VTA is required for induction of GABAergic STDP. The selective expression of this DA-dependent STDP in the VTA presents an important synaptic learning mechanism that can be targeted by drugs of abuse or stress to alter DA signaling within VTA DA circuits and significantly impact reward-related behavioral learning.

## Data Availability

All relevant data is contained within the manuscript: all datasets (GENERATED/ANALYZED) for this study are included in the manuscript.

## Author Contributions

FN designed the experiments. LL, MD and FN performed electrophysiology experiments, analyzed the data and prepared the figures. LL and FN wrote the manuscript.

## Conflict of Interest Statement

The authors declare that the research was conducted in the absence of any commercial or financial relationships that could be construed as a potential conflict of interest.

## Abbreviations

AKAP: A kinase anchoring protein
CaN: Calcineurin
DA: dopamine
D1R: dopamine D1-like receptor
D2R: dopamine D2-like receptor
IPSC: Inhibitory postsynaptic current
LTD: long-term depression
LTP: Long-term potentiation
LFS: Low frequency stimulation
NAc: Nucleus accumbens
bAPs: propagating action potentials
PKA: protein kinase A
RMTg: rostromedial tegmental area
STDP: Spike-timing dependent plasticity
SNc: Substantia pars compacta
VTA: Ventral tegmental area.

## References

[B1] Authement M. E. Kodangattil J. N. Gouty S. Rusnak M. Symes A. J. Cox B. M.. (2015). Histone deacetylase inhibition rescues maternal deprivation-induced GABAergic metaplasticity through restoration of AKAP signaling. Neuron 86, 1240–1252. 10.1016/j.neuron.2015.05.024 26050042

[B2] Barrot M. Sesack S. R. Georges F. Pistis M. Hong S. Jhou T. C. (2012). Braking dopamine systems: a new GABA master structure for mesolimbic and nigrostriatal functions. J. Neurosci. 32, 14094–14101. 10.1523/jneurosci.3370-12.2012 23055478 PMC3513755

[B3] Beckstead M. J. Ford C. P. Phillips P. E. Williams J. T. (2007). Presynaptic regulation of dendrodendritic dopamine transmission. Eur. J. Neurosci. 26, 1479–1488. 10.1111/j.1460-9568.2007.05775.x 17822435 PMC3633601

[B4] Beckstead M. J. Grandy D. K. Wickman K. Williams J. T. (2004). Vesicular dopamine release elicits an inhibitory postsynaptic current in midbrain dopamine neurons. Neuron 42, 939–946. 10.1016/j.neuron.2004.05.019 15207238

[B5] Beier K. T. Steinberg E. E. Deloach K. E. Xie S. Miyamichi K. Schwarz L.. (2015). Circuit architecture of VTA dopamine neurons revealed by systematic input-output mapping. Cell 162, 622–634. 10.1016/j.cell.2015.07.015 26232228 PMC4522312

[B6] Bradberry C. W. Roth R. H. (1989). Cocaine increases extracellular dopamine in rat nucleus accumbens and ventral tegmental area as shown by *in vivo* microdialysis. Neurosci. Lett. 103, 97–102. 10.1016/0304-3940(89)90492-8 2779859

[B7] Brzosko Z. Schultz W. Paulsen O. (2015). Retroactive modulation of spike timing-dependent plasticity by dopamine. Elife 4:e09685. 10.7554/elife.09685 26516682 PMC4626806

[B8] Cameron D. L. Williams J. T. (1993). Dopamine D1 receptors facilitate transmitter release. Nature 366, 344–347. 10.1038/366344a0 8247128

[B9] Campbell A. D. Kohl R. R. McBride W. J. (1996). Serotonin-3 receptor and ethanol-stimulated somatodendritic dopamine release. Alcohol 13, 569–574. 10.1016/s0741-8329(96)00069-9 8949951

[B10] Caporale N. Dan Y. (2008). Spike timing-dependent plasticity: a Hebbian learning rule. Annu. Rev. Neurosci. 31, 25–46. 10.1146/annurev.neuro.31.060407.125639 18275283

[B12] Dacher M. Gouty S. Dash S. Cox B. M. Nugent F. S. (2013). A-kinase anchoring protein-calcineurin signaling in long-term depression of GABAergic synapses. J. Neurosci. 33, 2650–2660. 10.1523/jneurosci.2037-12.2013 23392692 PMC6619159

[B11] Dacher M. Nugent F. S. (2011). Morphine-induced modulation of LTD at GABAergic synapses in the ventral tegmental area. Neuropharmacology 61, 1166–1171. 10.1016/j.neuropharm.2010.11.012 21129388

[B13] Dan Y. Poo M. M. (2006). Spike timing-dependent plasticity: from synapse to perception. Physiol. Rev. 86, 1033–1048. 10.1152/physrev.00030.2005 16816145

[B14] Feldman D. E. (2012). The spike-timing dependence of plasticity. Neuron 75, 556–571. 10.1016/j.neuron.2012.08.001 22920249 PMC3431193

[B15] Foncelle A. Mendes A. Jedrzejewska-Szmek J. Valtcheva S. Berry H. Blackwell K. T.. (2018). Modulation of spike-timing dependent plasticity: towards the inclusion of a third factor in computational models. Front. Comput. Neurosci. 12:49. 10.3389/fncom.2018.00049 30018546 PMC6037788

[B16] Ford C. P. Gantz S. C. Phillips P. E. Williams J. T. (2010). Control of extracellular dopamine at dendrite and axon terminals. J. Neurosci. 30, 6975–6983. 10.1523/jneurosci.1020-10.2010 20484639 PMC2883253

[B17] Johnson S. W. North R. A. (1992). Two types of neurone in the rat ventral tegmental area and their synaptic inputs. J. Physiol. 450, 455–468. 10.1113/jphysiol.1992.sp019136 1331427 PMC1176131

[B18] Kamikubo Y. Tabata T. Kakizawa S. Kawakami D. Watanabe M. Ogura A.. (2007). Postsynaptic GABAB receptor signalling enhances LTD in mouse cerebellar Purkinje cells. J. Physiol. 585, 549–563. 10.1113/jphysiol.2007.141010 17947316 PMC2375497

[B19] Klitenick M. A. Dewitte P. Kalivas P. W. (1992). Regulation of somatodendritic dopamine release in the ventral tegmental area by opioids and GABA: an *in vivo* microdialysis study. J. Neurosci. 12, 2623–2632. 10.1523/jneurosci.12-07-02623.1992 1319478 PMC6575858

[B20] Kodangattil J. N. Dacher M. Authement M. E. Nugent F. S. (2013). Spike timing-dependent plasticity at GABAergic synapses in the ventral tegmental area. J. Physiol. 591, 4699–4710. 10.1113/jphysiol.2013.257873 23897235 PMC3800449

[B21] Langlois L. D. Nugent F. S. (2017). Opiates and plasticity in the ventral tegmental area. ACS Chem. Neurosci. 8, 1830–1838. 10.1021/acschemneuro.7b00281 28768409 PMC5775906

[B22] Margolis E. B. Lock H. Chefer V. I. Shippenberg T. S. Hjelmstad G. O. Fields H. L. (2006a). Kappa opioids selectively control dopaminergic neurons projecting to the prefrontal cortex. Proc. Natl. Acad. Sci. U S A 103, 2938–2942. 10.1073/pnas.0511159103 16477003 PMC1413839

[B23] Margolis E. B. Lock H. Hjelmstad G. O. Fields H. L. (2006b). The ventral tegmental area revisited: is there an electrophysiological marker for dopaminergic neurons? J. Physiol. 577, 907–924. 10.1113/jphysiol.2006.117069 16959856 PMC1890372

[B24] Pan B. Hillard C. J. Liu Q. S. (2008). D2 dopamine receptor activation facilitates endocannabinoid-mediated long-term synaptic depression of GABAergic synaptic transmission in midbrain dopamine neurons via cAMP-protein kinase A signaling. J. Neurosci. 28, 14018–14030. 10.1523/jneurosci.4035-08.2008 19109485 PMC2656602

[B25] Pawlak V. Kerr J. N. (2008). Dopamine receptor activation is required for corticostriatal spike-timing-dependent plasticity. J. Neurosci. 28, 2435–2446. 10.1523/JNEUROSCI.4402-07.2008 18322089 PMC6671189

[B26] Pawlak V. Wickens J. R. Kirkwood A. Kerr J. N. (2010). Timing is not everything: neuromodulation opens the STDP gate. Front. Synaptic Neurosci. 2:146. 10.3389/fnsyn.2010.00146 21423532 PMC3059689

[B27] Pignatelli M. Bonci A. (2015). Role of dopamine neurons in reward and aversion: a synaptic plasticity perspective. Neuron 86, 1145–1157. 10.1016/j.neuron.2015.04.015 26050034

[B28] Polter A. M. Barcomb K. Tsuda A. C. Kauer J. A. (2018). Synaptic function and plasticity in identified inhibitory inputs onto VTA dopamine neurons. Eur. J. Neurosci. 47, 1208–1218. 10.1111/ejn.13879 29480954 PMC6487867

[B29] Rahman S. Zhang J. Corrigall W. A. (2003). Effects of acute and chronic nicotine on somatodendritic dopamine release of the rat ventral tegmental area: *in vivo* microdialysis study. Neurosci. Lett. 348, 61–64. 10.1016/s0304-3940(03)00723-7 12902018

[B30] Rice M. E. Cragg S. J. Greenfield S. A. (1997). Characteristics of electrically evoked somatodendritic dopamine release in substantia nigra and ventral tegmental area *in vitro*. J. Neurophysiol. 77, 853–862. 10.1152/jn.1997.77.2.853 9065854

[B31] Ruan H. Saur T. Yao W. D. (2014). Dopamine-enabled anti-Hebbian timing-dependent plasticity in prefrontal circuitry. Front. Neural Circuits 8:38. 10.3389/fncir.2014.00038 24795571 PMC4005942

[B32] Schilström B. Yaka R. Argilli E. Suvarna N. Schumann J. Chen B. T.. (2006). Cocaine enhances NMDA receptor-mediated currents in ventral tegmental area cells via dopamine D5 receptor-dependent redistribution of NMDA receptors. J. Neurosci. 26, 8549–8558. 10.1523/jneurosci.5179-05.2006 16914681 PMC6674361

[B33] Shen W. Flajolet M. Greengard P. Surmeier D. J. (2008). Dichotomous dopaminergic control of striatal synaptic plasticity. Science 321, 848–851. 10.1126/science.1160575 18687967 PMC2833421

[B34] Shin J. H. Adrover M. F. Alvarez V. A. (2017). Distinctive modulation of dopamine release in the nucleus accumbens shell mediated by dopamine and acetylcholine receptors. J. Neurosci. 37, 11166–11180. 10.1523/jneurosci.0596-17.2017 29030431 PMC5688525

[B35] Xu T. X. Yao W. D. (2010). D1 and D2 dopamine receptors in separate circuits cooperate to drive associative long-term potentiation in the prefrontal cortex. Proc. Natl. Acad. Sci. U S A 107, 16366–16371. 10.1073/pnas.1004108107 20805489 PMC2941310

[B36] Zhang J. C. Lau P. M. Bi G. Q. (2009). Gain in sensitivity and loss in temporal contrast of STDP by dopaminergic modulation at hippocampal synapses. Proc. Natl. Acad. Sci. U S A 106, 13028–13033. 10.1073/pnas.0900546106 19620735 PMC2713390

[B37] Zhang T. A. Placzek A. N. Dani J. A. (2010). *In vitro* identification and electrophysiological characterization of dopamine neurons in the ventral tegmental area. Neuropharmacology 59, 431–436. 10.1016/j.neuropharm.2010.06.004 20600174 PMC2946471

